# CCDC65 as a new potential tumor suppressor induced by metformin inhibits activation of AKT1 via ubiquitination of ENO1 in gastric cancer

**DOI:** 10.7150/thno.54961

**Published:** 2021-07-13

**Authors:** Tongyuan Deng, Peng Shen, Aimin Li, Ziyan Zhang, Huiling Yang, Xiaojie Deng, Xuemei Peng, Zhe Hu, Zibo Tang, Jiahao Liu, Rentao Hou, Zhen Liu, Weiyi Fang

**Affiliations:** 1 Cancer Center, Integrated Hospital of Traditional Chinese Medicine, Southern Medical University, 510315 Guangzhou, China.; 2 Cancer Institute, Southern Medical University, 510515 Guangzhou, China.; 3 Department of Oncology, Nanfang Hospital, Southern Medical University, 510515 Guangzhou, China.; 4 Key Laboratory of Protein Modification and Degradation, School of Basic Medical Sciences, Affiliated Cancer Hospital and Institute of Guangzhou Medical University, 511436 Guangzhou, China.; 5 School of Pharmacy, Guangdong Medical University, 523808 Dongguan, China.; These authors contributed equally: Tongyuan Deng, Peng Shen, Aimin Li, Ziyan Zhang.

**Keywords:** Gastric cancer, CCDC65, ENO1, AKT1, Metformin

## Abstract

The coiled-coil domain containing protein members have been well documented for their roles in many diseases including cancers. However, the function of the coiled-coil domain containing 65 (CCDC65) remains unknown in tumorigenesis including gastric cancer.

**Methods:** CCDC65 expression and its correlation with clinical features and prognosis of gastric cancer were analyzed in tissue. The biological role and molecular basis of CCDC65 were performed via in vitro and in vivo assays and a various of experimental methods including co-immunoprecipitation (Co-IP), GST-pull down and ubiquitination analysis et al. Finally, whether metformin affects the pathogenesis of gastric cancer by regulating CCDC65 and its-mediated signaling was investigated.

**Results**: Here, we found that downregulated CCDC65 level was showed as an unfavourable factor in gastric cancer patients. Subsequently, CCDC65 or its domain (a.a. 130-484) was identified as a significant suppressor in GC growth and metastasis in vitro and in vivo. Molecular basis showed that CCDC65 bound to ENO1, an oncogenic factor has been widely reported to promote the tumor pathogenesis, by its domain (a.a. 130-484) and further promoted ubiquitylation and degradation of ENO1 by recruiting E3 ubiquitin ligase FBXW7. The downregulated ENO1 decreased the binding with AKT1 and further inactivated AKT1, which led to the loss of cell proliferation and EMT signal. Finally, we observed that metformin, a new anti-cancer drug, can significantly induce CCDC65 to suppress ENO1-AKT1 complex-mediated cell proliferation and EMT signals and finally suppresses the malignant phenotypes of gastric cancer cells.

**Conclusion:** These results firstly highlight a critical role of CCDC65 in suppressing ENO1-AKT1 pathway to reduce the progression of gastric cancer and reveals a new molecular mechanism for metformin in suppressing gastric cancer. Our present study provides a new insight into the mechanism and therapy for gastric cancer.

## Introduction

Gastric cancer is one of the most common malignancies and has become the third major cause of cancer mortality in China 1. Although many advancements in diagnoses and treatments for GC have been made, most patients are found to be in the advanced stage at the time of initial diagnosis and the 5-year survival rate of those patients is quite low, which is partially because of the lack of knowledge on molecular mechanism of GC progression and effective therapeutic targets 2-4. To this end, it is extremely urgent to elucidate more effective biomarkers and targets as underlying GC development and progression.

Coiled-Coil Domain Containing 65 (CCDC65), located on human chromosome 12q13.12, has an open reading frame of 1455 nucleotides encoding a protein of 484 amino acids and belongs to coiled-coil domain-containing (CCDC) protein family 5. The CCDC proteins exert different functions according to their highly versatile folding motif, which is found in many proteins like motor and skeletal proteins and involved in protein refolding and molecular recognition systems 6. More than 3000 genes contain the coiled-coil domain, but their functions are currently unclear in many cases. Previous studies have shown that CCDC genes are associated with many diseases, including cancers, metabolic diseases, hearing loss, epigenetic disease, mitochondrial disease and heart disease 7, 8. The CCDC65 plays a vital role in the assembly of the nexin-dynein regulatory complex and mutations in this gene can result in primary ciliary dyskinesia 9-11. In addition, genetic or epigenetic alterations of CCDC genes associated with human cancers have been reported, including CCDC67 in papillary thyroid carcinoma, CCDC19 (NESG1) in lung cancer, CCDC50 in mantle cell lymphoma and chronic lymphocytic leukemia, and CCDC8 in breast cancer 12-15. However, few studies have investigated the role and mechanism of CCDC65 in cancers.

Metformin is one of the most commonly used non-insulin drugs for patients with type 2 diabetes mellitus (T2D) 16. However, in recent years a growing body of research has revealed that metformin has been reported to have anticancer activity in vivo and in vitro 17. The potential antitumor effects of metformin in GC have also been reported. Several studies showed that metformin can significantly inhibits the proliferation of gastric cancer cells in vitro and in vivo by inducing cell cycle arrest and inhibits EMT of gastric cancer by β-catenin pathway 18-21. The molecular mechanism underlying the suppression of gastric cancer by metformin still remains fully understood.

In this study, we examined the expression of CCDC65 in GC tissues and found that CCDC65 was decreased in GC and negatively correlated with clinical stage, lymph node metastasis, distant metastasis and prognosis. Notably, the in vitro and in vivo experiments revealed that CCDC65 and its 130-484aa domain strongly suppressed the proliferation and metastasis of GC. Mechanistically, CCDC65 induced by metformin suppresses the cell growth and metastasis of GC, acting through the repression of AKT1 signaling pathway via promoting FBXW7-mediated ubiquitination and degradation of ENO1. Taken together, CCDC65 may serve as a promising diagnostic marker and therapeutic target for GC patients.

## Methods

### Cell culture and sample collection

Human gastric cancer cell lines (AGS and SGC7901) were obtained from the Cell Bank of the Chinese Academy of Sciences. HEK293T cells were obtained from the Cancer Research Institute of Southern Medical University. GC cell lines were cultured in RPMI 1640 medium (Invitrogen) supplemented with 10% fetal bovine serum (Biowest, Loire Valley, France), while HEK293T cells were grown in Dulbecco's modified Eagle's medium (Invitrogen) with 10% fetal bovine serum (Biowest, Loire Valley, France). All cell lines were confirmed to be free of mycoplasma contamination and were grown in a humidified air atmosphere containing 5% CO_2_ at 37 °C. Nine surgical resected fresh primary GC tissues and paired normal gastric tissues were obtained from the Cancer Center, Integrated Hospital of Traditional Chinese Medicine, Southern Medical University (Guangdong, China). Clinical processes were approved by the Ethics Committees of the Integrated Hospital of Traditional Chinese Medicine of Southern Medical University, and patients provided informed consent. One hundred and eighty-seven paraffin-embedded primary GC specimens and one hundred and seventy-three paraffin-embedded adjacent nontumor specimens were included in our study. All specimens had a confirmed pathological diagnosis. Clinical data were obtained from the medical records of the patients who had not received preoperative radiation, chemotherapy, or biotherapy.

### Lentivirus production and infection

The human CCDC65 gene was inserted into the GV219 vector to create CCDC65 lentivirus (LV-CCDC65) (GeneChem, Shanghai, China). A lentivirus with the empty GV219 vector (LV-NC) was constructed as a control (GeneChem, Shanghai, China). The GC cells were infected with LV-NC or LV-CCDC65, and polyclonal cells with green fluorescent protein signals were selected for further experiments (Figure S1C-D).

### Cell transfection

The siRNAs for CCDC65, ENO1 and FBXW7 were designed and synthesized by RiboBio (Guangzhou, China; Table S2). The full length and the truncated mutant plasmids of CCDC65, ENO1 and AKT1 were purchased from GeneChem (Shanghai, China). GC cells were plated onto cell culture plate or dish (Nest, Biotech, China) at 30%-50% confluence and then transfected 24 h later using Lipofectamine 2000 (Invitrogen Biotechnology, USA). Plasmids or siRNA were transfected at a working concentration according to the manufacturer's protocol. 48-72 h after transfection, cells were collected for further experiments.

### Metformin treatment

Metformin (Selleck, Cambridge, UK) was resuspended in PBS (150 mM) prior to the experiment and stored at -20 °C. Cytotoxicity assay of metformin was detected by the 3-(4,5-dimethylthiazol-2-yl)-2,5-diphenyltetrazolium bromide (MTT) assay. Cells were seeded in 96-well plates in 100 µL culture medium at 5 × 10^3^ cells per well. Once attached, cells were treated with different concentrations of metformin and incubated at 37 °C in 5% CO_2_ for 48 h. The absorbance was measured by spectrophotometry following the manufacturer's protocol.

### RNA isolation, RT-PCR and qRT-PCR

Total RNA from the cultured cells or tissues was extracted by using the TRIzol reagent kit (Takara, Dalian, China) and was reversely transcribed into complementary DNA (cDNA) by using reverse transcription reagents (TaKaRa, Shiga, Japan) according to the manufacturer's instructions. The Bio-Rad T100 system was applied for RT-PCR. QRT-PCR was performed with the SYBR Premix ExTaq (TaKaRa, Shiga, Japan). The cDNA was used as a template to amplify with specific primers (Table S3). Bio-Rad CFX96 detection system was applied for qRT-PCR. The fold changes of genes were calculated by using the 2^-ΔΔCt^ method.

### Western blotting

Proteins were collected from cells in lysis buffer and were used for quantification with a BCA protein assay kit (Thermo Scientific, Waltham, MA, USA). The proteins were subjected to SDS-PAGE electrophoresis and transferred onto polyvinylidene fluoride membranes, which were immunoprobed with the corresponding primary antibodies. Western blotting (WB) was performed according to a previous description 22. Primary antibodies including anti-CCDC65, ENO1, AKT1, p-AKT1 (Ser473), FBXW7, E-cadherin, N-cadherin, Vimentin, CCND1, P21, GST, His, Myc, Flag, HA, GAPDH, β-tubulin and β-actin are listed in Supplementary (Table S4). Images were captured with ChemiDocTM CRSþ Molecular Imager (Bio-Rad, USA).

### The cycloheximide (CHX) chase assay

Cycloheximide (Selleck, Cambridge, UK) was resuspended in DMSO (200 mM) prior to the experiment and stored at -20 °C. Cells were seeded in 6-well plates in 2 mL culture medium. Once attached, cells were incubated with 50 µg/mL CHX at 37 °C in 5% CO_2_ for different duration. Subsequently, the cells were collected and further analyzed by using western blot analysis.

### Immunofluorescence

The cells grown on laser confocal petri dish were rinsed with PBS and fixed with 4% paraformaldehyde for 15 min at room temperature. Subsequently, the cells were permeabilized with 0.1% Triton X-100 for 30 min. After three washes with PBS, the samples were blocked with normal goat serum for 1 h at room temperature and incubated with different antibodies in PBS overnight at 4 °C. After three washes with PBS, the cells were incubated with the secondary antibody in the dark at room temperature for 1 h. The cell nuclei were stained with DAPI (blue), and the cells were then observed under a fluorescence microscope (ZEISS, Germany) at the same exposure. Primary antibodies included anti-CCDC65, ENO1 and AKT1. Antibodies are listed in Supplementary (Table S4).

### MTT assay

Cell viability was assessed by using the MTT assay. Exponentially growing cells were seeded into 96 well plates in 100 µL medium and incubated overnight to allow adherence. Cell viability was measured using MTT (Sigma Aldrich, MO, USA). The absorbance was measured by spectrophotometry following the manufacturer's protocol.

### Colony formation assay

Cells were seeded in 6-well culture plates at 100 cells per well. The culture medium was replaced every 24 h. After incubation for 14 days, cells were washed twice with PBS and stained with hematoxylin solution after fixation with paraformaldehyde for 15 min. The number of colonies containing 50 cells was counted under a microscope.

### EdU incorporation assay

EdU incorporation was performed by using Apollo567 In Vitro Imaging Kit (RiboBio, Guangzhou, China) according to the manufacturer's protocol. Cells were incubated with 10 μM EdU for 2 h before fixation with 4% paraformaldehyde. After permeabilization with 0.3% Triton X-100, the cells were stained with Apollo fluorescent dyes. Cell nuclei were stained with DAPI.

### Migration, invasion and wound healing assays

Migration and invasion were assessed using the Transwell (Millipore, Bedford, MA, USA) assay. For the invasion assay, the Transwell was pre-coated with matrigel (BD Biosciences Pharmingen, USA) for 30 min. Cells cultured in serum free medium were added to the upper chamber, and the bottom chamber was filled with culture media containing 10% FBS. After incubation, the migrated cells were fixed, stained, photographed and quantified by counting cells in three random fields per filter. Cells were plated in 6-well plates. After cells reached confluence, artificial wound tracks were scratched using a pipette tip. Wound closure was visualized at 0 h and 48 h by using a microscope.

### Animal experiments

Animal experiments complied with the requirements of the Animal Research Committee of the Academic Medical Centre at Southern Medical University and the international guidelines for animal care and maintenance. SGC7901 cells (5 × 10^6^ cells per mouse) were subcutaneously injected into the right flanks of 4-week-old BALB/c female nude mice. The tumor volumes were measured with a vernier calliper every 3 days. On day 28 after inoculation, the mice were humanely sacrificed according to institutional ethics guidelines, and tumors were harvested and weighted. For in vivo metastasis assays, the pulmonary metastasis model was used. GC cells (2 × 10^6^ cells per mouse) were injected into the caudal vein of nude mice. On day 40 after caudal vein injection, optical and pathological images were collected.

Subcutaneous tumor xenograft model was also constructed to assess the treatment effect of si-NC, si-CCDC65, normal saline (NS), metformin alone or in combination with si-CCDC65. The SGC7901 cells were injected subcutaneously into the right flank of 4-week-old female Balb/c nude mice. When the xenografts reached a mean diameter of 6 mm, animals were treated with an intraperitoneal injection once a day by metformin (250 mg/kg) or an intratumoral multi-point injection every 3 days with complexes of 12 µg siRNA, si-CCDC65 (CCDC65 siRNA treatment group) or si-NC (Non-target siRNA treatment group) together with 6 µL Entranster-in vivo (Engreen Biosystem Co, Ltd, Beijing, China) respectively as previous studies described 23, 24. The tumor volumes and the weight of mice were measured every 3 days. Animals were sacrificed 34 days after treatment.

### Immunohistochemistry

Paraffin-embedded tissues sections (4-μm-thick) were deparaffinised and dehydrated, subjected to antigen retrieval, blocked endogenous nonspecific antigen and peroxidase activity with goat serum and 3% H_2_O_2_ and incubated overnight at 4 °C with primary antibodies (Table S4). After three times washes with PBS, the sections were incubated with HRP-conjugated secondary antibody and visualized using DAB substrate (Maixin, Fuzhou, China). The IHC results for tissues were scored by two independent observers according to both the percentage of positively stained cells (scored from 0 to 3) and the staining intensity (scored from 0 to 3).^25^ The final staining scores were obtained by multiplying the intensity and the extent scores. For statistical analysis, tumor tissues with final staining scores of <6 indicated low expression, and ≥ 6 indicated high expression.

### Coimmunoprecipitation

Coimmunoprecipitation was carried out using a Pierce Co-Immunoprecipitation kit (Thermo Scientific, MA, USA) according to the manufacturer's instructions. Briefly, total proteins were extracted and quantified. A total of 1 mg of protein was incubated with 5 μg of primary antibodies (Table S4) or IgG overnight at 4 °C. After elution, immune complexes were subjected to mass spectrometry, Western blot analysis and coomassie brilliant blue staining. Anti-IgG was used as a negative control.

### GST pull-down

Fusion proteins GST-CCDC65, His-ENO1 and His-FBXW7 were constructed by inserting the coding region of human CCDC65, ENO1 and FBXW7 into pET-28a and pGEX-6P-1, respectively. These recombinant plasmids were transformed into E. Coli DH5a and then induced by IPTG (Sangon, shanghai, China). The soluble lysates were prepared by using protease and phosphatase inhibitors. The fusion proteins were purified by using glutathione sepharose. His-ENO1 and His-FBXW7 fusion protein was prepared. For GST pull-down, the total protein was incubated with GST-fusion protein or GST-control at 4 °C with shaking overnight before incubation with glutathione-sepharose beads for 2 h. Beads were washed several times with binding buffer and boiled in SDS loading buffer at 100 °C for 10 min. WB was used to detect bound proteins.

### Statistical analyses

SPSS 25.0 (SPSS, Chicago, USA) was used for statistical analyses. Data are presented as the mean ± SD from at least three independent experiments. Statistical significance was determined by Student's t-test or one-way ANOVA with Tukey's multiple comparison tests. Correlations between two continuous variables were examined by Pearson's test. Survival analysis were analyzed by the Kaplan-Meier method and log-rank tests. P value < 0.05 indicated statistical significance (* P < 0.05, ** P < 0.01).

## Results

### Decreased CCDC65 expression correlates with poor prognosis of GC patients

**Figure 1:**
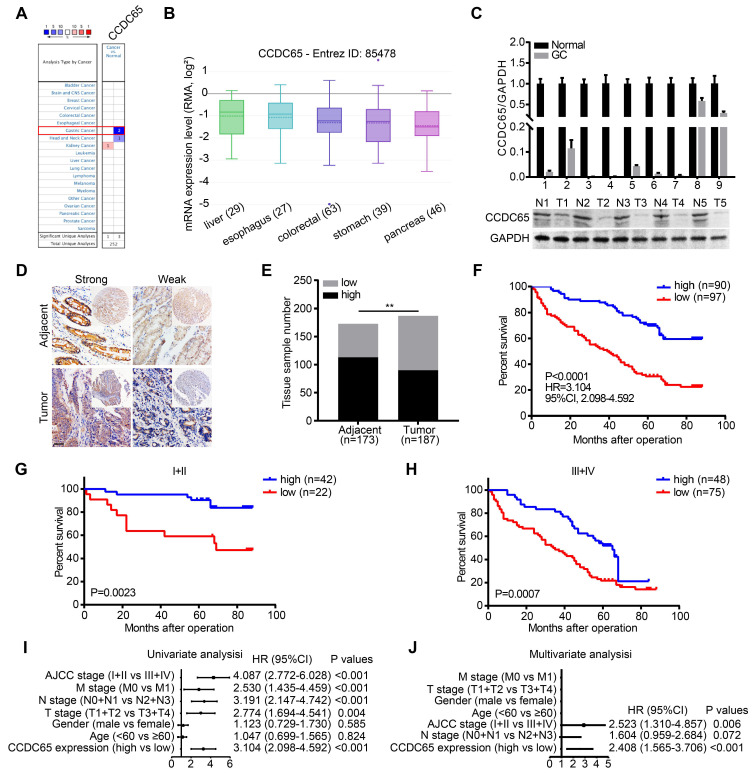
** Decreased CCDC65 expression correlates with poor prognosis in gastric cancer. (A)** The transcription level of CCDC65 in different types of cancers, analyzing by Oncomine. **(B)** The expression of CCDC65 in gastric cancer cell lines, analyzing by CCLE. **(C)** The expression level of CCDC65 was analyzed by qRT-PCR and WB in gastric cancer (GC) tissues and normal adjacent tissues (N). **(D-E)** The expression of CCDC65 in tumor and adjacent samples were monitored by immunohistochemistry (IHC) on a tissue array. The anti-CCDC65 antibody was employed for IHC. IHC scoring was performed blind, prior to association with clinical data. **(F)** Kaplan-Meier analysis of CCDC65 expression in 187 gastric cancer patients those were subdivided into two groups. **(G-H)** Stratified survival analysis of CCDC65 expression in clinical stage I-II and III-IV gastric cancer patients. **(I-J)** Univariate and multivariate analyses of different clinicopathological features in human gastric cancer patients.

To identify the potential role of CCDC65 in cancers, we compared the transcriptional levels of CCDC65 in cancers with those in normal samples by using ONCOMINE databases. The mRNA expression levels of CCDC65 were significantly decreased in GC patients in two datasets (Figure 1A). By assembling the Cancer Cell Line Encyclopedia (CCLE), we found that CCDC65 were lowly expressed in most cell lines of GC (Figure 1B). To further determine the expression of CCDC65 in GC, we detected the expression of CCDC65 in 9 fresh GC specimens and adjacent tissues by quantitative PCR (qRT-PCR) and western blotting (WB). The results showed that CCDC65 expression was significantly lower in GC tissues compare to adjacent tissues (Figure 1C). We then detected CCDC65 expression in 187 GC tissues and 173 corresponding para-carcinoma tissues by immunohistochemical staining (Figure 1D). Consistent with the above results, CCDC65 expression was downregulated in GC tissues compared with that in para-carcinoma tissues (Figure 1E). Survival analysis further indicated that GC patients with elevated CCDC65 expression had longer survival times than patients with low CCDC65 levels (Figure 1F). In addition, the stratified analysis of GC patients in clinical stage I-II and III-IV showed that low expression of CCDC65 was significantly associated with shorter survival time (Figure 1G-H). To further analyze the correlation between the level of CCDC65 with clinicopathological features, those GC samples were divided into high CCDC65 group and low CCDC65 group based on the median expression of CCDC65. We found that CCDC65 expression levels were significantly correlated with T stages, lymphatic metastasis, distant metastasis and clinical stage (Table S1). Importantly, univariate and multivariate analyses suggested that CCDC65 expression was an independent indicator for the overall survival of GC patients (Figure 1I-J). In summary, CCDC65 was an appropriate diagnostic and prognostic marker for GC.

### CCDC65 suppresses GC cell proliferation and tumorigenicity

**Figure 2:**
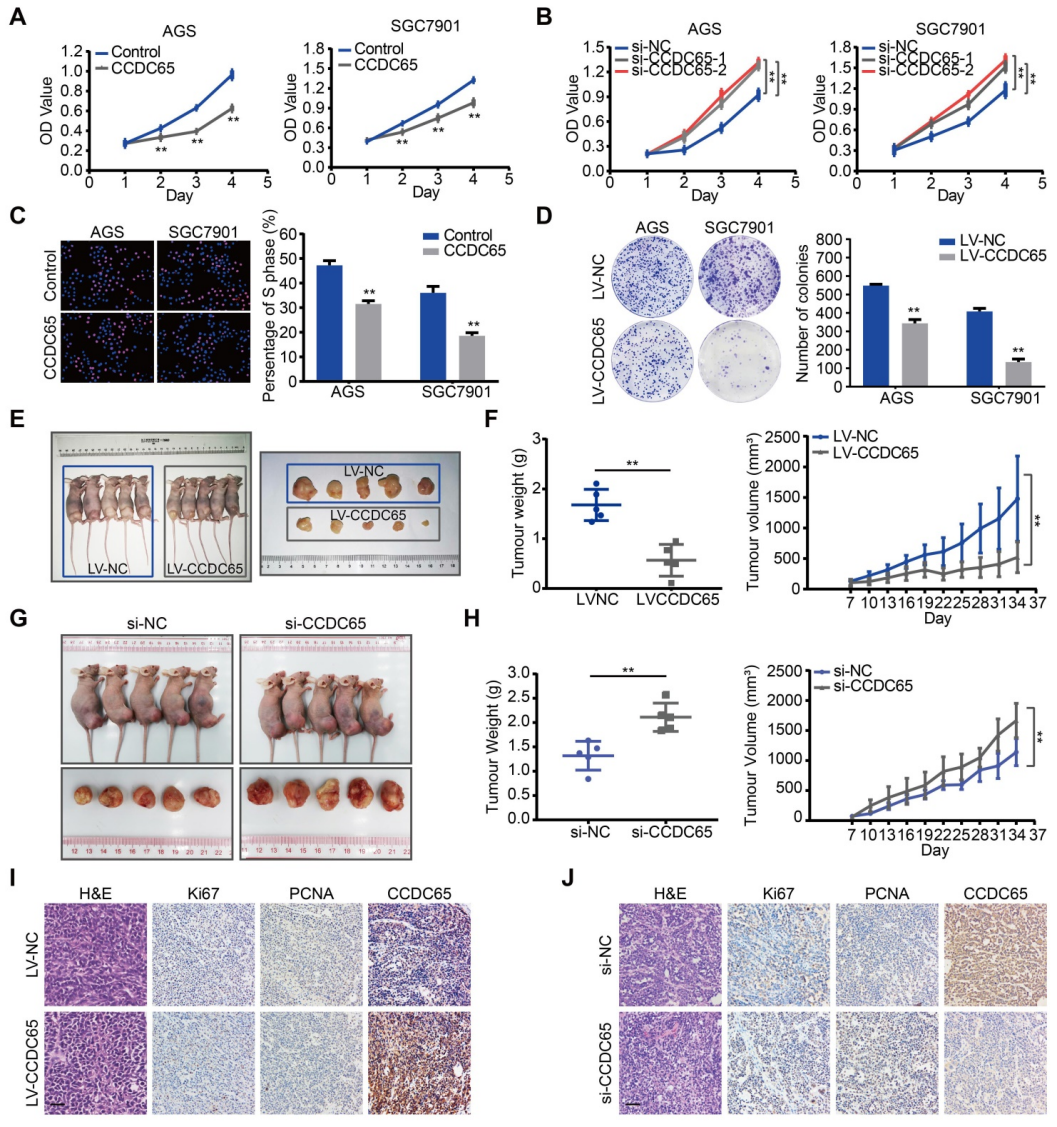
**CCDC65 suppresses gastric cancer growth in vitro and in vivo. (A-B)** CCDC65 overexpression markedly inhibited cell viability while CCDC65 knockdown promoted cell viability in AGS and SGC7901 cells by MTT assay. Student's t test. Mean ± SD, * p < 0.05; ** p < 0.01. **(C-D)** EdU incorporation assays (Scale bar: 100 μm) and colony formation assays of CCDC65-overexpressing AGS and SGC7901 cells and corresponding control cells. **(E)** The in vivo effect of CCDC65 was evaluated in xenograft mouse model bearing tumors originating from AGS cells. Each group contained 5 mice. **(F)** The growth curve and weight of xenograft were obtained in nude mice using stably expressing vector control or CCDC65 cells. **(G)** The in vivo effect of si-CCDC65 was evaluated in xenograft mouse model bearing tumors originating from SGC7901 cells. Each group contained 5 mice. **(H)** The growth curve and weight of xenograft were obtained in nude mice which were injected control or si-CCDC65 every 3 days. **(I-J)** Representative H&E as well as IHC staining of primary tumor tissues are shown. Scale bar, 50 μm.

To explore the biological role for CCDC65 in GC, CCDC65 plasmid and lentivirus were introduced into AGS and SGC7901 cell lines. The expression of CCDC65 was verified by qRT-PCR analysis (Figure S1A-B). We also used 3 siRNAs to knock down the expression of CCDC65 in AGS and SGC7901 cells and then found that the si-1 and si-2 siRNA had the best knockdown efficiency (Figure S1E). Therefore, we used them for the follow-up experiments. Overexpression of CCDC65 significantly inhibited cell viability in AGS and SGC7901 cells (Figure 2A). Inversely, CCDC65 knockdown obviously enhanced cell viability (Figure 2B). Further, through Edu incorporation assays (Figure 2C) and colony formation assays (Figure 2D) we observed that CCDC65 overexpression slowed GC cells proliferation and colony formation.

To investigate the biological functions of CCDC65 in vivo, we established stably CCDC65-overexpressed cell lines. These cells were subcutaneously implanted in nude mice. The growth and weight of xenografts was significantly decreased in CCDC65-overexpressed group compared with that in control group (Figure 2E-F). Moreover, to further examine the effect of CCDC65 knockdown in vivo, GC cells were inoculated subcutaneously into nude mice. At 7 days after the inoculation, we injected si-NC or si-CCDC65 into the developed tumors every 3 days. As a result, a significant enhancement of tumor growth and weight was observed in the group injected with si-CCDC65 (Figure 2G-H). By immunohistochemistry (IHC), the xenografts displayed the higher CCDC65 expression levels, the lower expression of Ki67 (Figure S6A) and proliferating cell nuclear antigen (PCNA) in CCDC65-overexpressed group compared to those in the control group (Figure 2I), while downregulated expression of CCDC65, elevated expression of Ki67 (Figure S6B) and PCNA in si-CCDC65 group compared with the control group (Figure 2J). These above results suggest that CCDC65 overexpression exerts a significant inhibitory effect on GC proliferation and tumorigenicity.

### CCDC65 suppresses GC cell migration, invasion and metastasis

**Figure 3:**
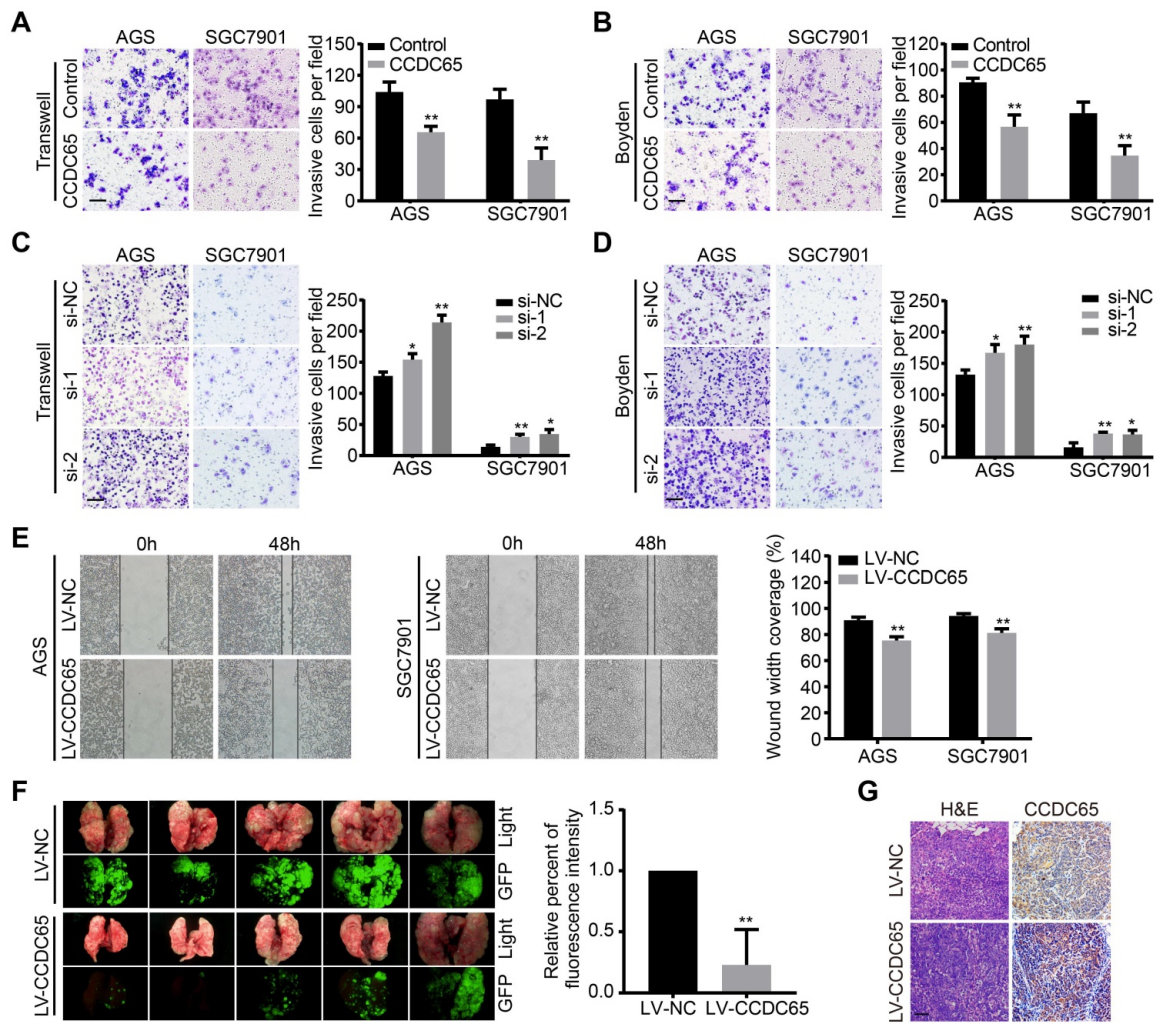
** CCDC65 suppresses gastric cancer metastasis in vitro and in vivo. (A-D)** Transwell assay, boyden assay and **(E)** wound-healing assays evaluated the migration and invasion of gastric cancer cells treated with CCDC65 plasmid, si-CCDC65, CCDC65 lentivirus and corresponding control group. **(F)** A pulmonary metastasis model was adopted to evaluate the effect of CCDC65 on metastasis (n = 5). **(G)** Representative H&E as well as IHC staining of pulmonary metastatic nodules are shown. Scale bar, 50 μm.

To examine the effect of CCDC65 on GC cell migration and invasion, a transwell apparatus and boyden chamber coated with matrigel were used. Compared with the control group, CCDC65-overexpressed group reduced cell migration and invasion in transwell (Figure 3A) and boyden (Figure 3B) assays. On the contrary, CCDC65 knockdown promoted cell migration and invasion in both AGS and SGC7901 cell groups compared to their respective control cells (Figure 3C-D). Furthermore, we found that CCDC65-overexpressed group obviously inhibited wound healing in wound-healing assay (Figure 3E). To investigate the antitumor effect of CCDC65 in vivo, we established pulmonary metastasis mouse model. Compared to the control group, less metastatic nodules and elevated expression of CCDC65 were detected in the CCDC65-overexpressed group as shown in our fluorescent and histopathologic assays (Figure 3F-G). These results suggest that CCDC65 decreases the metastatic potential of GC cells.

### CCDC65 suppresses GC proliferation and metastasis via the AKT1 signaling pathway

**Figure 4:**
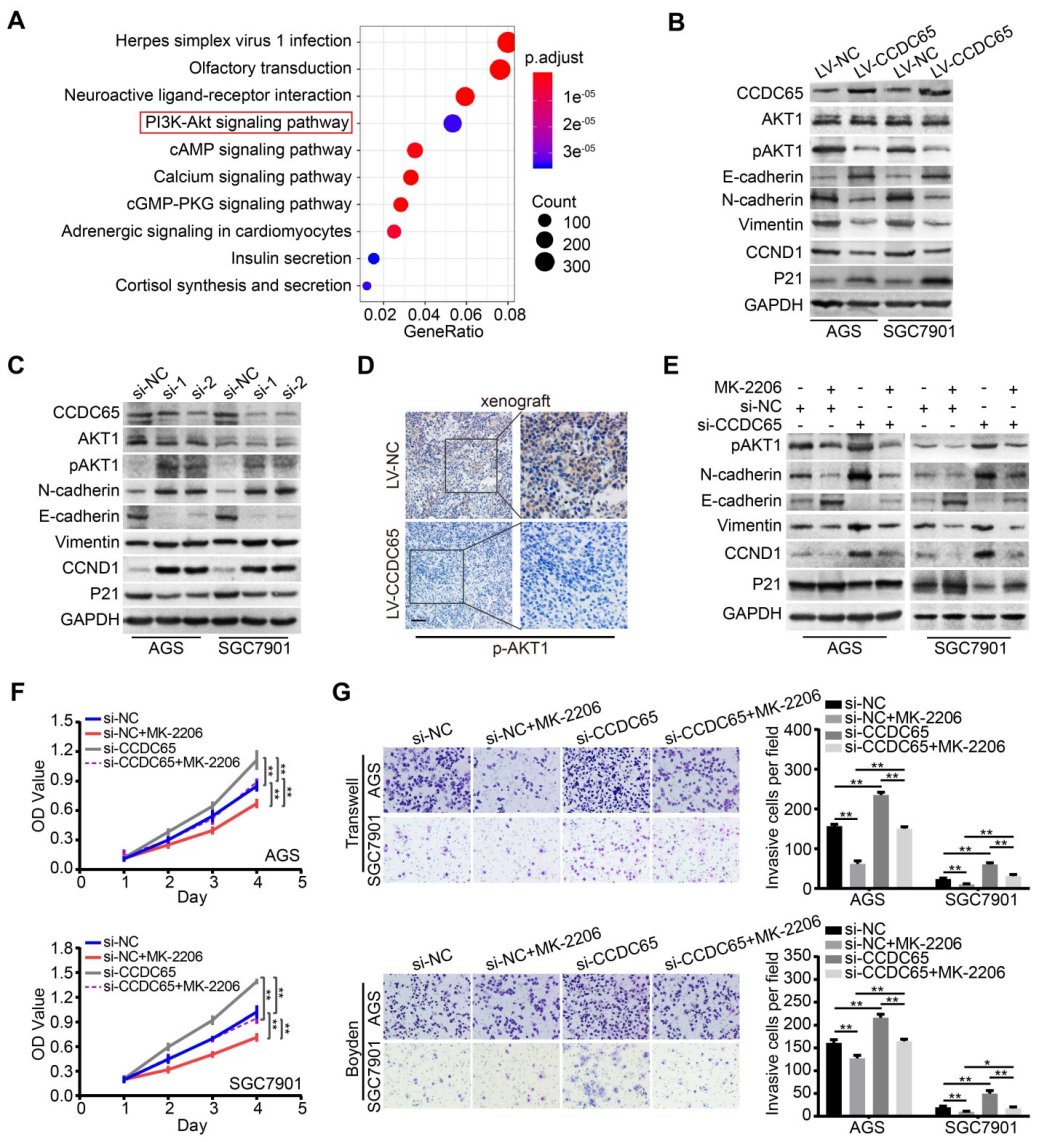
** CCDC65 involves in regulating AKT1 signaling pathway. (A)** Schematic diagram of the potential downstream pathways of CCDC65 with the TCGA gastric cancer RNAseq (IlluminaHiSeq; n = 415) data set. **(B-C)** Expression levels of CCDC65, AKT1, p-AKT1 (ser473), E-cadherin, N-cadherin, Vimentin, CCND1, P21 were detected following transfection with CCDC65 lentivirus and siRNA. GAPDH was used as a loading control. **(D)** IHC staining of p-AKT1 in primary tumor tissues. Scale bar, 50 μm. **(E)** Expression levels of p-AKT1 (Ser473), N-cadherin, E-cadherin, Vimentin, CCND1 and P21 in si-NC, si-NC+MK-2206, si-CCDC65, si-CCDC65+MK-2206 groups. **(F-G)** MTT assays, transwell and boyden assays evaluated the cell viability and metastasis of si-NC, si-NC+MK-2206, si-CCDC65, si-CCDC65+MK-2206 groups.

The critical role of CCDC65 in tumor progression motivated us to identify the mechanism by which CCDC65 suppresses cell growth and metastasis. To further investigate the downstream pathway involved in CCDC65-mediated tumor suppression, we analyzed the genes related to CCDC65 expression in 415 GC patients using The Cancer Genome Atlas (TCGA) database. We found that 13305 genes were significantly correlated with CCDC65. Further, Kyoto Encyclopedia of Genes and Genomes (KEGG) pathway analysis was conducted based on these genes, revealing the top 10 enrichment-related pathways (Figure 4A). As the PI3K-AKT signaling pathway plays an important role in the regulation of tumor progression 26, 27, we then analyzed the relationship between CCDC65 and PI3K-AKT signaling pathway and found that CCDC65 significantly suppressed the levels of p-AKT1 (Ser473), but not total AKT1 protein (Figure 4B). In addition, we found that CCDC65 overexpression downregulated N-cadherin, Vimentin and CCND1, but elevated E-cadherin and P21 expression (Figure 4B). Inversely, CCDC65 knockdown brought completely different results (Figure 4C). IHC in xenograft tumor tissues confirmed that p-AKT1 (Ser473) was suppressed by CCDC65 overexpression (Figure 4D). Using AKT1 specific inhibitor MK-2206 into si-CCDC65 GC cells neutralized si-CCDC65-modulated upregulation of p-AKT1 (Ser473), N-cadherin, Vimentin and CCND1 and downregulation of E-cadherin and P21 (Figure 4E). Cell proliferation, migration and invasion were inhibited in MTT assays, transwell and boyden assays (Figure 4F-G) after MK-2206 treatment in si-CCDC65 cells. The above results suggest that CCDC65 attenuates cell proliferation and metastasis through modulating AKT1 pathway.

### CCDC65 interacts with ENO1 to suppress the proliferation and metastasis of GC

**Figure 5:**
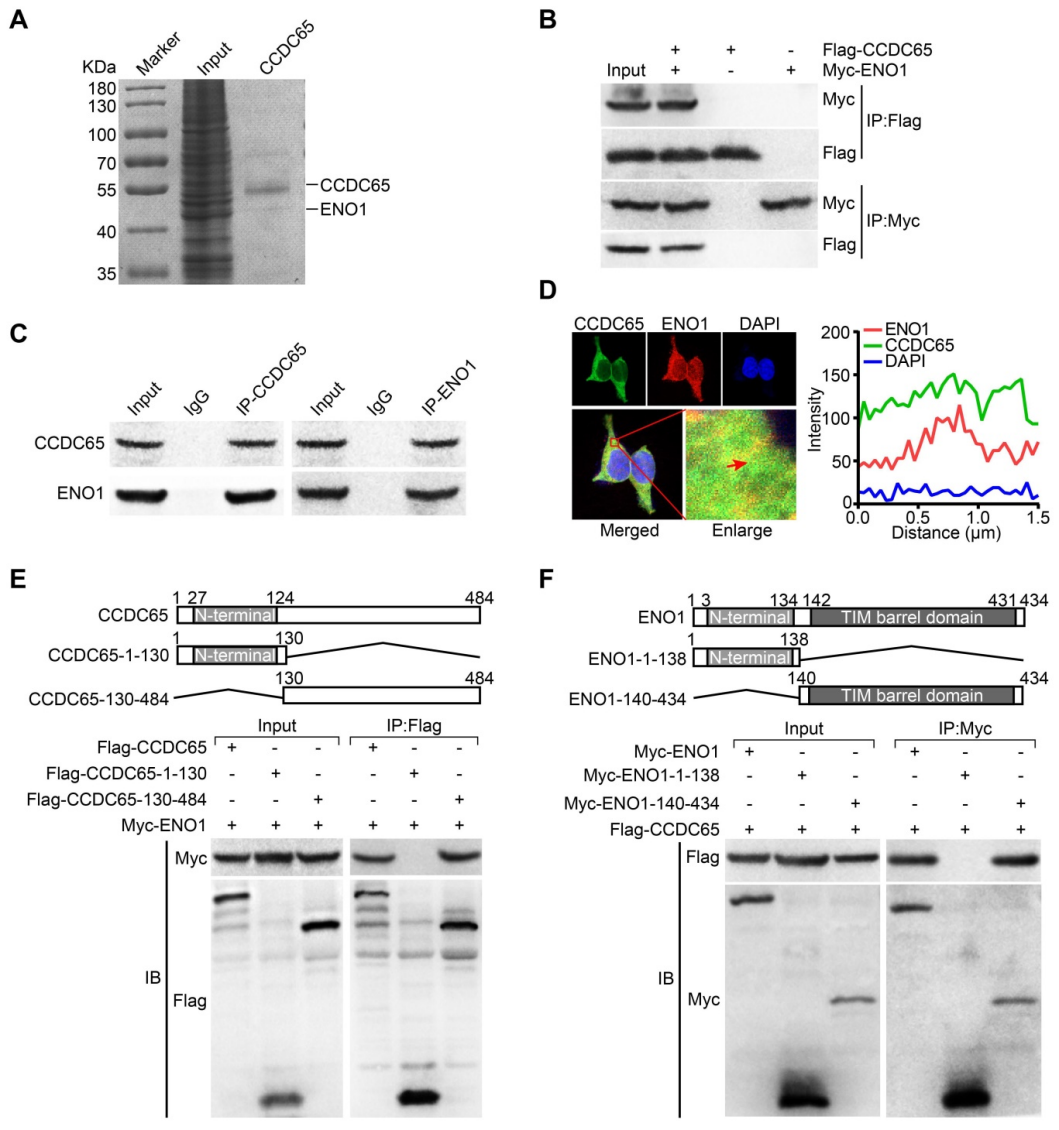
** CCDC65 interacts with ENO1. (A)** Coomassie brilliant blue staining showed the proteins that interacted with CCDC65 in AGS cells and the molecular weight of CCDC65 and ENO1. **(B)** Co-IP detected the interaction of exogenous CCDC65 and ENO1. **(C)** Co-IP detected the interaction of endogenous CCDC65 and ENO1. **(D)** Colocalization of CCDC65 and ENO1 in HEK293T cells by immunofluorescence staining. **(E)** N-terminal deletion mutant domain (Flag-CCDC65-130-484) of CCDC65 is important for interaction with ENO1. The mutants of CCDC65 were transfected into HEK293T cells and analyzed by immunoprecipitation using anti-Flag antibody. **(F)** The C-terminal TIM barrel domain (Myc-ENO1-140-434) of ENO1 is required for its interaction with CCDC65. The mutants of ENO1 were transfected into HEK293T cells and analyzed by immunoprecipitation using anti-Myc antibody.

To explore the precise molecular mechanisms of CCDC65 repressing AKT1 pathway, Co-IP combined with mass spectrometry was used in AGS cells. By screening the predicted interacting proteins of mass spectrometry, we found a candidate interaction protein ENO1 (47 kDa band; Figure 5A). Many studies have reported that ENO1 plays a promoting role in different tumors including GC by regulating the AKT pathway 28, 29. Further, we investigated whether CCDC65 indeed has an interaction with ENO1, the exogenous and endogenous co-immunoprecipitation (Co-IP) demonstrated that CCDC65 interacted with ENO1 (Figure 5B-C). Moreover, immunofluorescence showed that CCDC65 and ENO1 proteins mainly colocalized in cytoplasm (Figure 5D). To reveal the exact interaction domains between CCDC65 and ENO1, Co-IP analyses were tested by using 2 truncated mutants of CCDC65 and 2 truncated mutants of ENO1. And then we found that the N-terminal deletion mutant (Flag-CCDC65-130-484) strongly bound to Myc-ENO1 but not C-terminal deletion mutant (Flag-CCDC65-1-130) (Figure 5E). The C-terminal TIM barrel domain (Myc-ENO1-140-434) co-precipitated with the wild type (WT) CCDC65 protein, but not the C-terminal deletion mutant (Myc-ENO1-1-138) (Figure 5F).

To determine the tumorigenicity of CCDC65 domains, two CCDC65 truncated mutants including N-terminal deletion mutant (Flag-CCDC65-130-484) and C-terminal deletion mutant (Flag-CCDC65-1-130) were expressed in GC cells. N-terminal deletion mutant (Flag-CCDC65-130-484) showed the capabilities to suppress cell proliferation and metastasis but the C-terminal deletion mutant (Flag-CCDC65-1-130) lacked these properties (Figure S2A-B). Moreover, N-terminal deletion mutant (Flag-CCDC65-130-484) inhibited ENO1 and p-AKT1 (Ser473) in comparison with the control group, whereas C-terminal deletion mutant (Flag-CCDC65-1-130) lost this effect (Figure S2C). Collectively, these data suggest that CCDC65 exerts its effects in GC progression through interacting with ENO1 by its C-terminal domain (a.a. 130-484).

### CCDC65 recruits E3 ubiquitin ligase FBXW7 to regulate ENO1 protein stability

**Figure 6:**
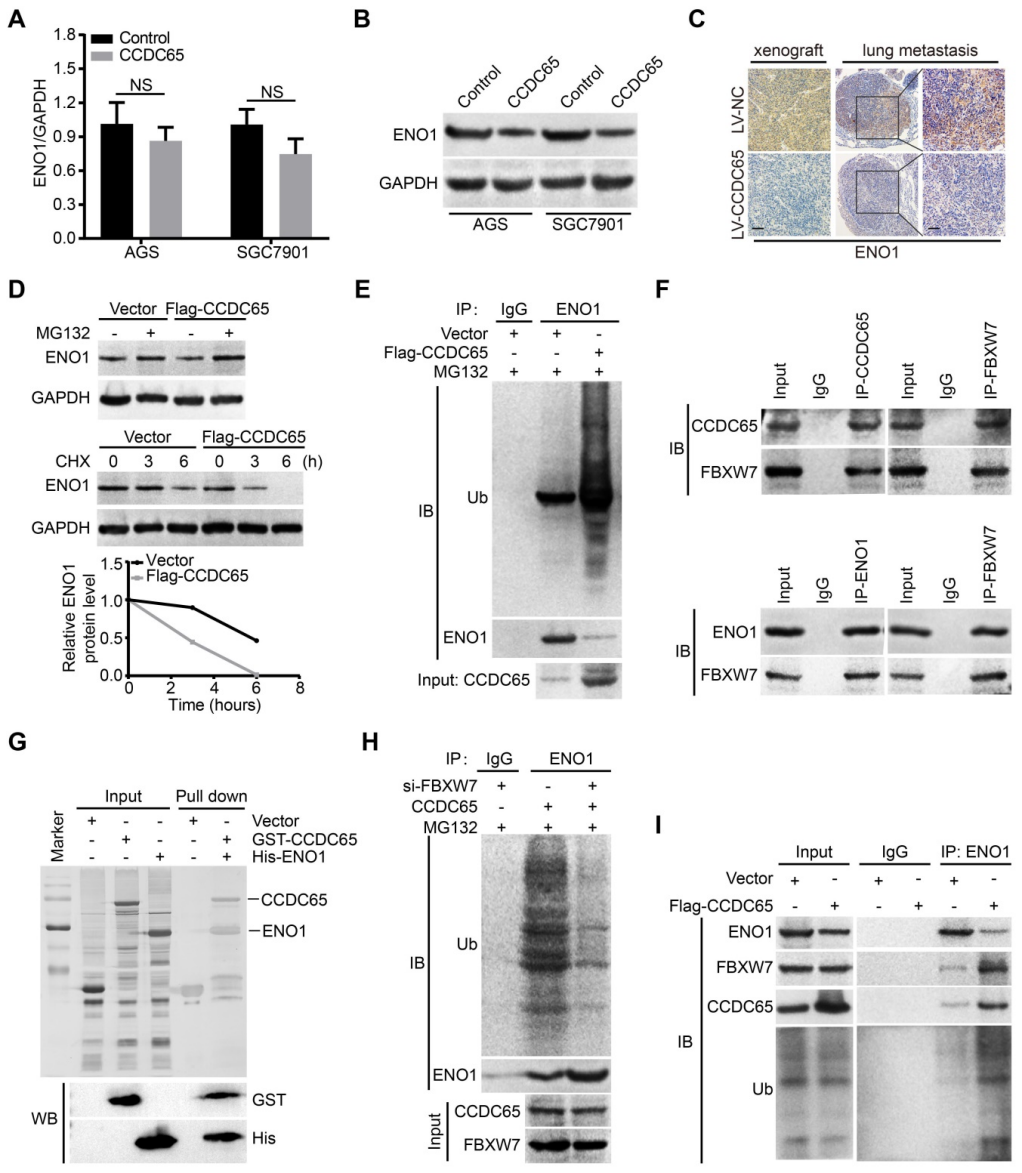
** CCDC65 recruits FBXW7 to promotes the degradation of ENO1. (A)** The expression of ENO1 mRNA was detected by qRT-PCR after CCDC65 overexpression, normalized to GAPDH. Student's t test, mean ± SD, * p < 0.05; ** p < 0.01. **(B)** The protein expression of ENO1 were detected by WB after CCDC65 overexpression. **(C)** IHC staining of ENO1 in primary tumor tissues and pulmonary metastatic nodules were shown. Scale bar, 50 μm. **(D)** WB was used to detect the effects of DMSO or MG132 treatment and CHX treatment for different duration on the stability of ENO1 protein in the control and CCDC65 overexpression groups. **(E)** Co-IP detected the effects of CCDC65 overexpression on protein stability of ENO1 in GC cells. **(F)** Co-IP detected the interaction of CCDC65 and ENO1 with FBXW7. **(G)** GST-CCDC65 interacts with His-ENO1 in vitro by GST pull-down assay. **(H)** Co-IP detected the effects of FBXW7 knockdown on protein stability of ENO1 in CCDC65 overexpression GC cells. **(I)** Co-IP was conducted to identify the function of CCDC65 overexpression on the interplay among ENO1, FBXW7 and ubiquitin in GC cells.

Since CCDC65 interacted with ENO1, we tried to figure out whether CCDC65 is involved in regulating ENO1 expression. Interestingly, we found that the ENO1 protein level was markedly downregulated after CCDC65 overexpression in WB and IHC assays, whereas the mRNA level of ENO1 was not affected (Figure 6A-C). Therefore, we suspected that the regulation of ENO1 by CCDC65 is unlikely at the transcriptional level. To test whether CCDC65 regulates ENO1 at the posttranscriptional level, cycloheximide (CHX) chasing assay and MG132 treatment was performed. The half-life of ENO1 protein was notably shortened and a remarkable accumulation of ENO1 proteins in CCDC65 overexpression cells with CHX and MG132 treatment (Figure 6D), suggesting that CCDC65 is involved in the regulation of ENO1 protein stability. Due to the fact that ubiquitin proteasome pathway is the common mechanism for protein degradation 30, we next determined whether CCDC65 induces ENO1 degradation by promoting its ubiquitination in GC cells. ENO1 was immunoprecipitated with specific anti-ENO1 antibodies, and its ubiquitination status was analyzed with anti-ubiquitin antibody. As expected, the overexpression of CCDC65 significantly increased the ubiquitination level of ENO1 (Figure 6E).

To further investigate the mechanism of CCDC65-activated degradation of ENO1 protein, we predicted that E3 ubiquitin ligase FBXW7 may interacted with ENO1 by using the BioGRID and HitPredict data sets. In addition, previous studies showed that FBXW7 mediates the ubiquitination level of ENO1 31, 32. Therefore, we detected whether the CCDC65-dependent degradation of ENO1 protein was indeed mediated by FBXW7. Endogenous Co-IP demonstrated that both CCDC65 and ENO1 interacted with FBXW7 (Figure 6F). Moreover, GST pull-down assays further validated that CCDC65 exhibited direct association with ENO1 (Figure 6G) and FBXW7 (Figure S3C). In addition, cycloheximide chasing assay and MG132 treatment showed that knockdown of FBXW7 abolished the effect of CCDC65 on the protein level of ENO1 (Figure S3A-B). Co-IP demonstrated that depletion of FBXW7 ameliorated the effect of CCDC65 on the ubiquitination and degradation of ENO1 (Figure 6H). Co-IP further implied that CCDC65 overexpression facilitated the capture of ubiquitin and FBXW7 by ENO1 (Figure 6I), while CCDC65 depletion triggered the dissociation of the ubiquitin ligase complex composed of ubiquitin, FBXW7 and ENO1 (Figure S3D). These results suggest that CCDC65 serving as a scaffold to recruits FBXW7 to regulate ENO1 protein stability.

To further examined whether ENO1 is involved in the inhibition of CCDC65 in GC, we found that transiently transfecting ENO1 plasmid into CCDC65-overexpressing cells enhanced cell proliferation based on MTT (Figure S4A). Furthermore, migration and invasion were enhanced in transwell and boyden assays (Figure S4B) after transfecting ENO1 plasmid into CCDC65-overexpressed cells. The overexpression of ENO1 significantly abrogated the CCDC65-mediated suppression of AKT1 pathway, N-cadherin, Vimentin, CCND1 and the stimulation of E-cadherin and P21 (Figure S4C), suggesting that ENO1 is involved in the CCDC65-regulated of AKT1 pathway in GC. Collectively, our data demonstrate that CCDC65 may serve as a docking platform facilitating the interaction of ENO1 with FBXW7 and degradation of ENO1, and the three proteins form a triple complex exerting an important role during GC progression.

### CCDC65 inhibits the ENO1-AKT1 interaction

**Figure 7:**
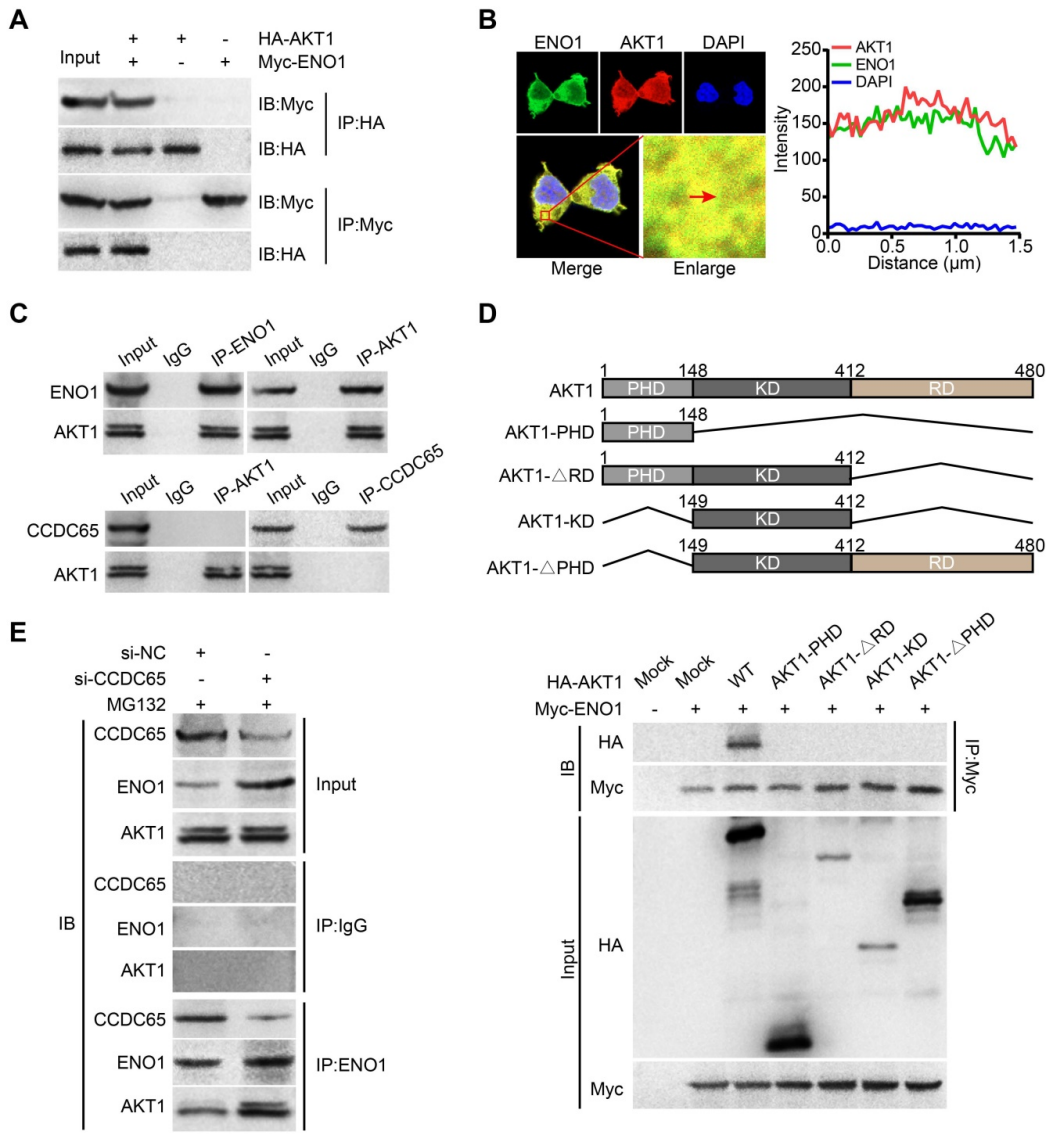
** ENO1 interacts with AKT1. (A)** Co-IP detected the interaction of exogenous ENO1 and AKT1. **(B)** Colocalization of ENO1 and AKT1 in HEK293T cells was detected by immunofluorescence staining. **(C)** Co-IP detected the interaction of endogenous ENO1 and CCDC65 with AKT1. **(D)** Schematic representations of AKT1 and its mutants. The full-length of AKT1 is important for interaction with ENO1. The mutants of AKT1 were transfected into HEK293T cells and analyzed by immunoprecipitation using anti-Myc antibody. **(E)** Co-IP detected the effects of si-CCDC65 on the interaction of ENO1 and AKT1 in GC cells.

Many studies had showed that ENO1 activates AKT pathway in tumor 28, 33. In our study, we found that CCDC65 can suppressed AKT1 pathway and reduced the protein level of ENO1. Therefore, we tried to figure out whether there is a direct correlation between ENO1 and AKT1 pathway. We searched two protein-protein interaction databases (BioGRID and HitPredict) and found that ENO1 might interact with AKT1. Exogenous Co-IP demonstrated that AKT1 interacted with ENO1 (Figure 7A). Immunofluorescence also showed that ENO1 and AKT1 proteins mainly colocalized in cytoplasm (Figure 7B). In addition, endogenous Co-IP showed that AKT1 interacted with ENO1, but not CCDC65 (Figure 7C). Moreover, a series of AKT1 truncated mutants were constructed and examined for the ENO1-binding ability. Our mutagenesis analysis revealed that only the full-length HA-AKT1 co-precipitated with the WT ENO1 protein, but not HA-AKT1^PHD^, HA-AKT1^∆RD^, HA-AKT1^KD^ and HA-AKT1^∆PHD^ (Figure 7D). In addition, when CCDC65 was knocked down, the interaction of the ENO1-AKT1 was more robustly (Figure 7E). These results demonstrate that the full-length of AKT1 is required for ENO1 interaction and CCDC65 interferes with the binding of ENO1 to AKT1.

### Metformin potentiates tumor suppression of CCDC65 in GC

**Figure 8:**
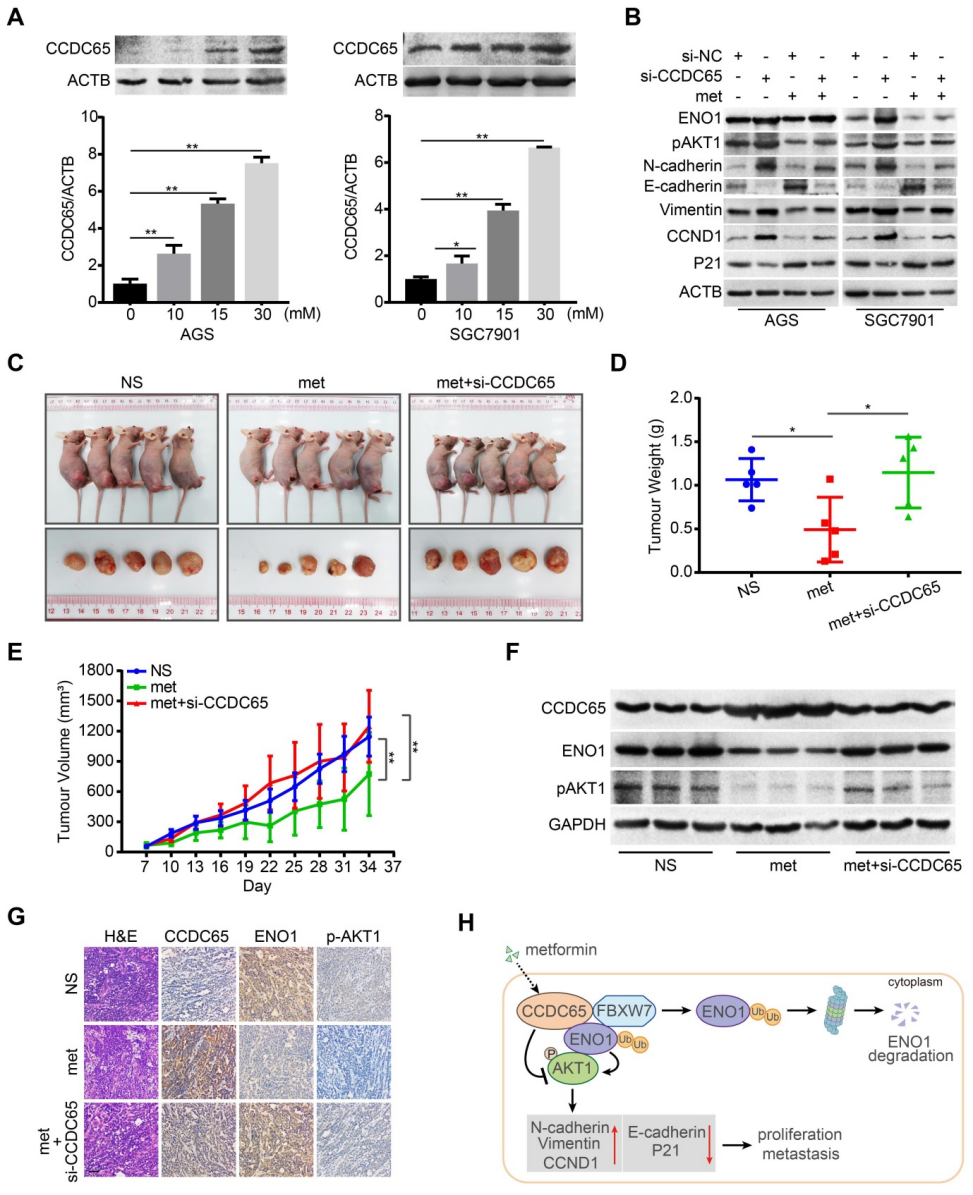
** Metformin potentiates tumor suppression of CCDC65 in vitro and in vivo. (A)** Metformin up-regulated CCDC65 expression in a dose-dependent manner in AGS and SGC7901 cells. **(B)** The expression of ENO1, p-AKT1 (ser473), N-cadherin, E-cadherin, Vimentin, CCND1 and P21 in si-NC, si-CCDC65, si-NC+metformin, si-CCDC65+metformin groups. **(C)** The in vivo effect of normal saline (NS), metformin (met) and metformin+si-CCDC65 groups was evaluated in xenograft mouse model bearing tumors originating from SGC7901 cells. Each group contained 5 mice. **(D-E)** The weight and growth curve of xenograft were obtained in nude mice treated with NS, met and metformin+si-CCDC65. **(F-G)** Comparisons of CCDC65, ENO1 and p-AKT1 in xenografts of NS, metformin and metformin+si-CCDC65 groups by western blot and IHC assays. **(H)** Working model of CCDC65 induced by metformin in inactivating AKT1 via the ubiquitination of ENO1 in gastric cancer.

There is plenty of evidence to demonstrate that metformin represents a promising anti-cancer drug in GC and could lead to a better survival rate 34, 35. In addition, previous studies have reported that metformin suppresses the expression of ENO1 in cancers 36, 37. We next investigated whether CCDC65 is involved in the metformin-induced suppression of GC cells. In the current study, metformin treatment resulted in a potent increase of CCDC65 expression, which occurred in a dose-dependent manner (Figure 8A). MTT assays indicated that, in combination with metformin treatment, si-CCDC65 group significantly restored the cell growth of GC in comparison to the si-CCDC65 group (Figure S5A). Transwell and boyden assays produced a similar result that si-CCDC65 combined with metformin enhanced migration and invasion of GC in comparison with the si-CCDC65 group (Figure S5B). Moreover, CCDC65 knockdown neutralized metformin-modulated downregulation of ENO1 and pAKT1, N-cadherin, Vimentin, CCND1 and upregulation of E-cadherin and P21 (Figure 8B).

To determine the potentiated effect of metformin on CCDC65 in vivo, we constructed subcutaneous tumor xenograft models. Among the three groups, metformin (250 mg/kg) group had the best inhibitory effect on xenograft growth, while metformin combined with si-CCDC65 group neutralized metformin-mediated inhibitory effect on growth and weight of xenograft (Figure 8C-E). However, the weight of mice had no significant differences among the three groups (Figure S5C). In addition, immunohistochemistry and WB assays showed that metformin increased expression of CCDC65 and si-CCDC65 abolished metformin-dependent suppression of ENO1 and pAKT1 (Figure 8F-G). These results suggest that CCDC65 plays a critical role in metformin-mediated suppression of GC by inactivating ENO1-AKT1 signal.

## Discussion

CCDC65 belongs to coiled-coil domain-containing protein family which acts as motor and skeletal protein, and is involved in protein refolding and molecular recognition systems as well as being associated with many diseases 38, 39. Many studies reported that CCDC genes associated with human cancers 40, 41. However, it is not clear whether CCDC65 has any role in cancers. Based on ONCOMINE and CCLE databases, we found that CCDC65 is down-regulated in GC. In the current study, CCDC65 was significantly decreased in GC patients, and was negatively correlated with clinical stage, pathological T stages, lymphatic and distant metastasis. Moreover, elevated CCDC65 expression had a good prognosis in GC patients. Subsequently, we observed that overexpression of CCDC65 inhibited GC cell proliferation, migration, invasion and metastasis in vitro and in vivo while CCDC65 knockdown had the opposite results. Thus, we demonstrated that CCDC65 functions as a potential tumor suppressor in GC.

AKT1, a serine/threonine kinase, is a vital component involved in the PI3K/AKT pathway 42-44. Dozens of studies have reported that AKT1 pathway is responsible for many cellular processes, such as proliferation, migration, invasion and metastasis 45-47. Additionally, the involvement of AKT1 signaling pathway in GC has been demonstrated by several studies 48, 49. Based on KEGG pathway analysis, the PI3K/AKT pathway was screened as a potential signal affected by CCDC65. In our study, we found that CCDC65 attenuates cell proliferation and metastasis through modulating AKT1 pathway. Moreover, si-CCDC65 mediated proliferation and EMT signaling were both blunted after AKT1 specific inhibitor MK-2206 using in GC cells.

To obtain further insight into the detailed molecular mechanism by which CCDC65 represses AKT1 pathway, we screened the interacting proteins of CCDC65. ENO1, an important rate-limiting metalloenzyme in glycolysis, is ubiquitous in most tissues, and mainly locates in cell cytoplasm and membrane 50, 51. Mounting evidence has demonstrated that ENO1 is a multifunctional protein depending on its cellular localization 29, 52. Moreover, a growing body of studies indicated that ENO1 contributed to tumor malignancy 53, 54. A previous study of our group has found that ENO1 promotes lung cancer progression through activating PI3K/AKT signaling pathway 55. In the present work, we identified that ENO1 is a directly associated protein of CCDC65. In addition, CCDC65 mediated the ubiquitination and degradation of ENO1. Previous studies have suggested that the ubiquitination of ENO1 was affected by E3 ubiquitin ligase FBXW7 31, 32. Interestingly, we further determined that CCDC65 forms a complex with both ENO1 and FBXW7, and markedly enhances their interaction as well as enhancing FBXW7-mediated ubiquitination and degradation of ENO1. Moreover, we found that ENO1 was involved in CCDC65-regulated of AKT1 pathway in GC. Therefore, the coordinated role of CCDC65-FBXW7-ENO1 complex is critical for CCDC65 in mediating the proliferation and EMT of GC.

Additionally, we found that ENO1 interacted with AKT1 and induced the phosphorylation of AKT1 at ser473, but AKT1 was not interacted with CCDC65 in GC. Moreover, we demonstrated that CCDC65 influenced the binding of ENO1 to AKT1. However, the analysis of AKT1 truncated mutants revealed that only the full-length AKT1 co-precipitated with the WT ENO1 protein, but not other truncated mutants. Hence, we suspect that AKT1 co-precipitated with ENO1 may depend on its unbroken protein space structure, which deserves to be further investigated. These findings suggested that CCDC65 suppresses AKT1 pathway via FBXW7-mediated ubiquitination and degradation of ENO1, providing a new mechanism by which CCDC65 modulates the expression of proteins associated with proliferation and metastasis in GC.

Previous studies demonstrated that metformin is a promising anti-cancer drug in GC 56-58. And many studies have reported that metformin suppresses expression of ENO1 37, 59. Thus, it is not clear whether metformin influences the CCDC65-mediated the downregulation of ENO1. In the current study, metformin treatment resulted in a potent increase of CCDC65 expression in a dose-dependent manner. Both the mRNA and protein level of CCDC65 were markedly changed after metformin treatment. Thus, we speculated that the regulation of CCDC65 by metformin is possible at the transcriptional level and some proteins such as transcription factors may participate in this regulation. Further study to investigate the molecules involved in the regulation of CCDC65 transcription is warranted. In addition, CCDC65 knockdown neutralized metformin-mediated suppression of GC cell proliferation, migration and invasion, the level of ENO1 proteins and the expression of molecules associated with proliferation and metastasis. Moreover, subcutaneous tumor xenograft models also showed that metformin induced the expression of CCDC65 while suppressed expression of ENO1, and si-CCDC65 treatment neutralized metformin-mediated inhibitory effect on GC. Hence, CCDC65 plays a vital role in the metformin-mediated suppression of GC.

## Conclusions

In summary, we have identified the powerful function of tumor suppressor CCDC65 in GC. CCDC65 induced by metformin suppresses the cell growth and metastasis of GC cells, acting through the repression of AKT1 signaling pathway via promoting FBXW7-induced ubiquitination and degradation of ENO1 (Figure 8H). Our study contributes to understanding the underlying role of metformin and its potential target protein CCDC65 in GC and highlights the biological and clinical bases for the potential use of CCDC65 as a novel diagnostic factor and useful therapeutic target for GC.

## Supplementary Material

Supplementary figures and tables.

## Abbreviations

GC: gastric cancer
CCDC65: coiled-coil domain containing 65
ENO1: alpha-Enolase
Co-IP: co-immunoprecipitation
TCGA: The Cancer Genome Atlas
PI3K: 1-Phosphatidylinositol-3-Kinase
AKT1: RAC-Alpha Serine/Threonine-Protein Kinase
CCLE: Cancer Cell Line Encyclopedia
qRT-PCR: Quantitative real-time PCR
PCNA: proliferating cell nuclear antigen
WB: western blot
IHC: Immunohistochemistry
HE: Haematoxylin and eosin
KEGG: Kyoto Encyclopedia of Genes and Genomes
Edu: 5-ethynyl-20-deoxyuridine
FBXW7: F-Box and WD Repeat Domain Containing 7
MTT: Methyl Thiazolyl Tetrazolium
GST: Glutathione S-transferase
