# Supplementary material for: CCDC65 as a new potential tumor suppressor induced by metformin inhibits activation of AKT1 via ubiquitination of ENO1 in gastric cancer
Source: Theranostics. 2021 Jul 13;11(16):8112–28. doi: 10.7150/thno.54961 (PMC8315052; doi:10.7150/thno.54961)
Supplement: Supplementary file 1 — Supplementary figures and tables. [file thnov11p8112s1.pdf]

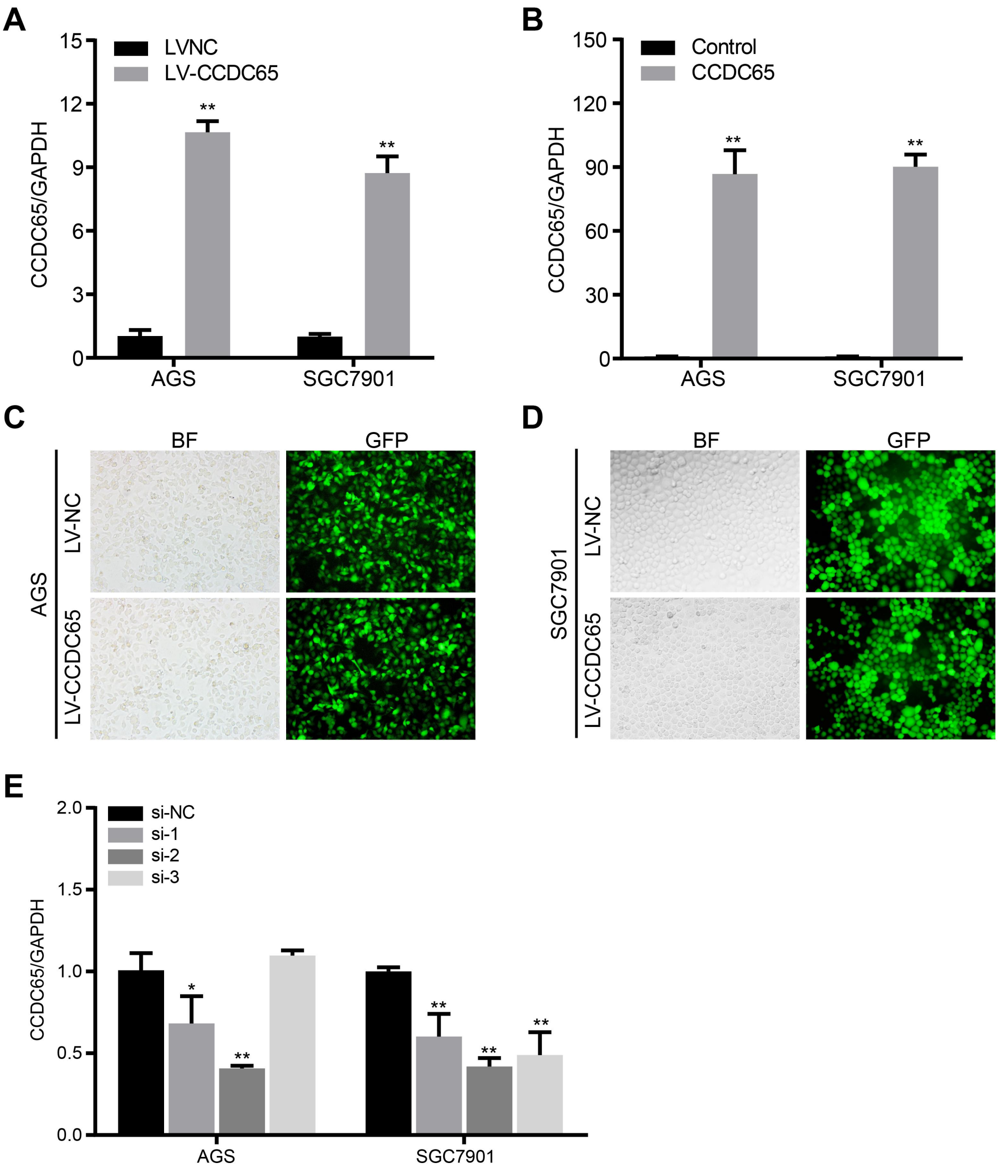

Supplementary Figure 1. The transfection efficiency of CCDC65 plasmid, lentivirus and siRNA. (A-B) The expression of CCDC65 in GC cells treated with CCDC65 lentivirus and plasmid. **(C-D)** The fluorescence of GFP was examined in LV-CCDC65 and LV-NC GC cells (scale bar: 50 μm). **(E)** The expression of CCDC65 in GC cells treated with CCDC65 siRNA and control was examined by QPCR.

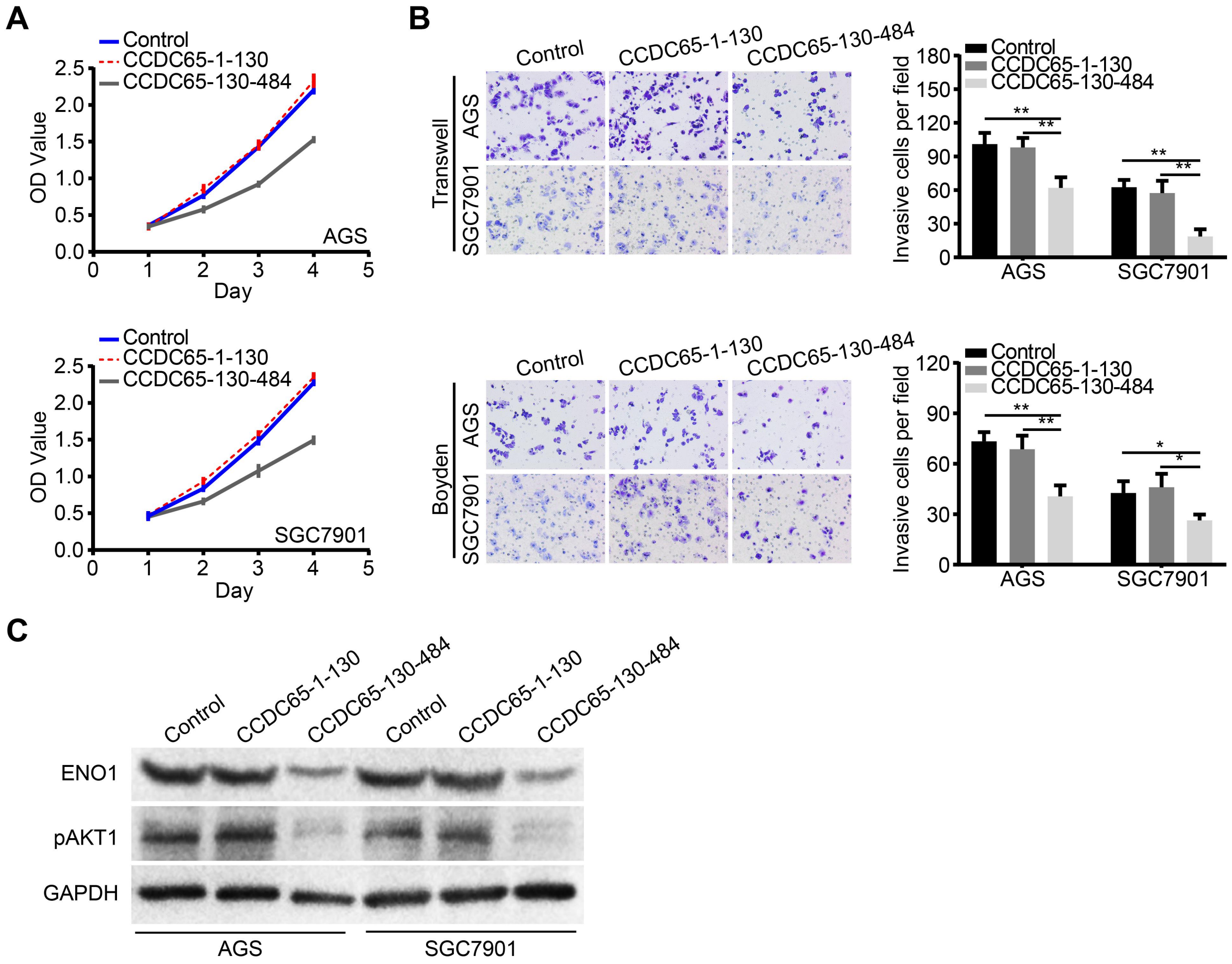

Supplementary Figure 2. The 130-484aa domain of CCDC65 suppressed cell proliferation and metastasis in GC. (A) MTT assays of two CCDC65 truncated mutants and control group in GC cells. All data are presented as the mean ± SD. **(B)** Transwell and boyden assay evaluated the migration and invasion of gastric cancer cells treated with two CCDC65 truncated mutants and control vector. Student's t test. **(C)** Expression levels of ENO1 and p-AKT1 (ser473) were detected following transfection with CCDC65 truncated mutants and control vector. GAPDH was used as a loading control.

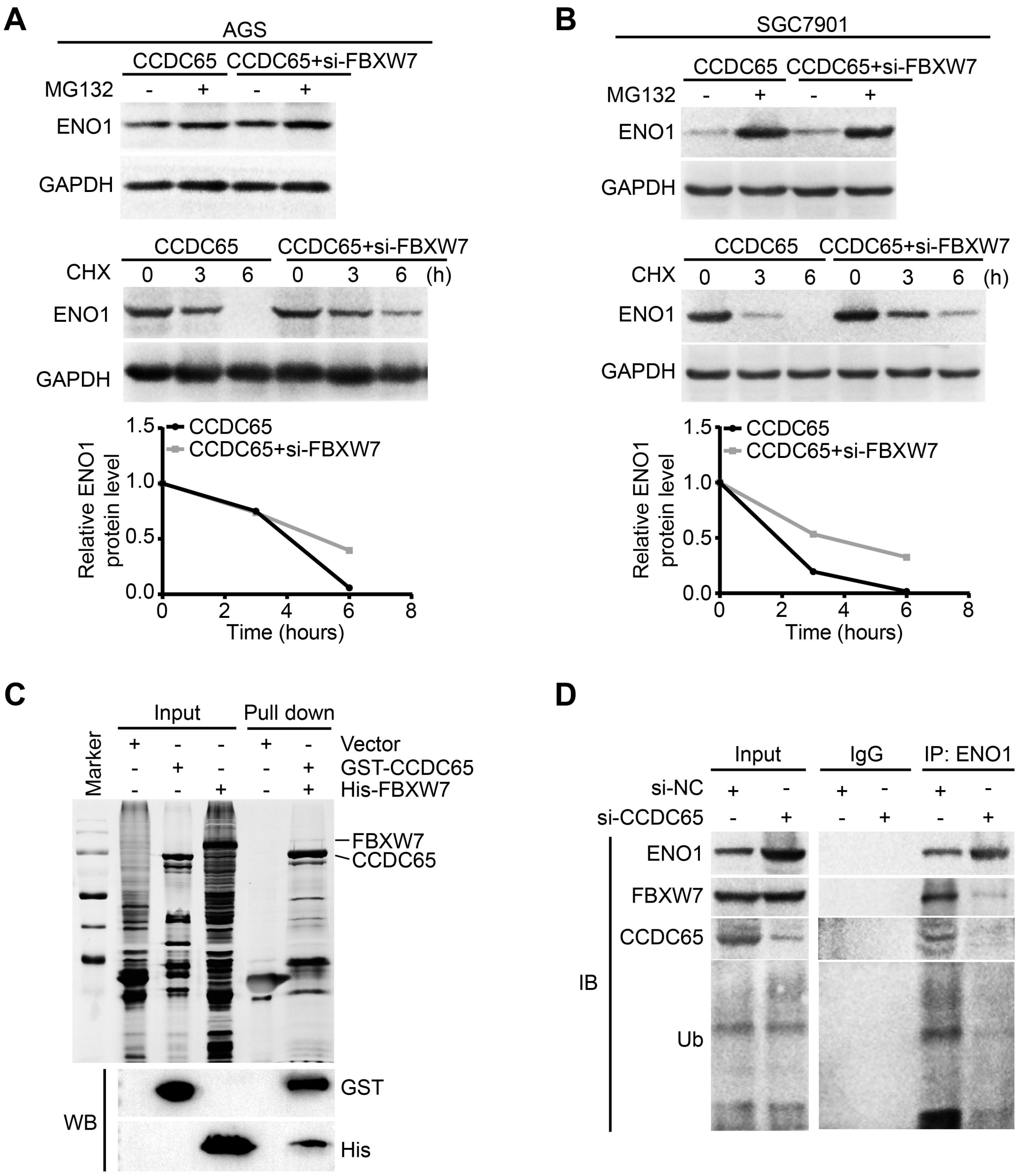

Supplementary Figure 3. CCDC65 recruits FBXW7 to regulate ENO1 protein stability. (A-B) WB was used to detect the effects of DMSO or MG132 treatment and CHX treatment for different duration on the stability of ENO1 protein in the CCDC65 and CCDC65+si-FBXW7 groups. **(C)** GST-CCDC65 interacts with His-FBXW7 in vitro by GST pull-down assay. **(D)** Co-IP was conducted to identify the function of CCDC65 knockdown on the interaction among ENO1, FBXW7 and ubiquitin in GC cells.

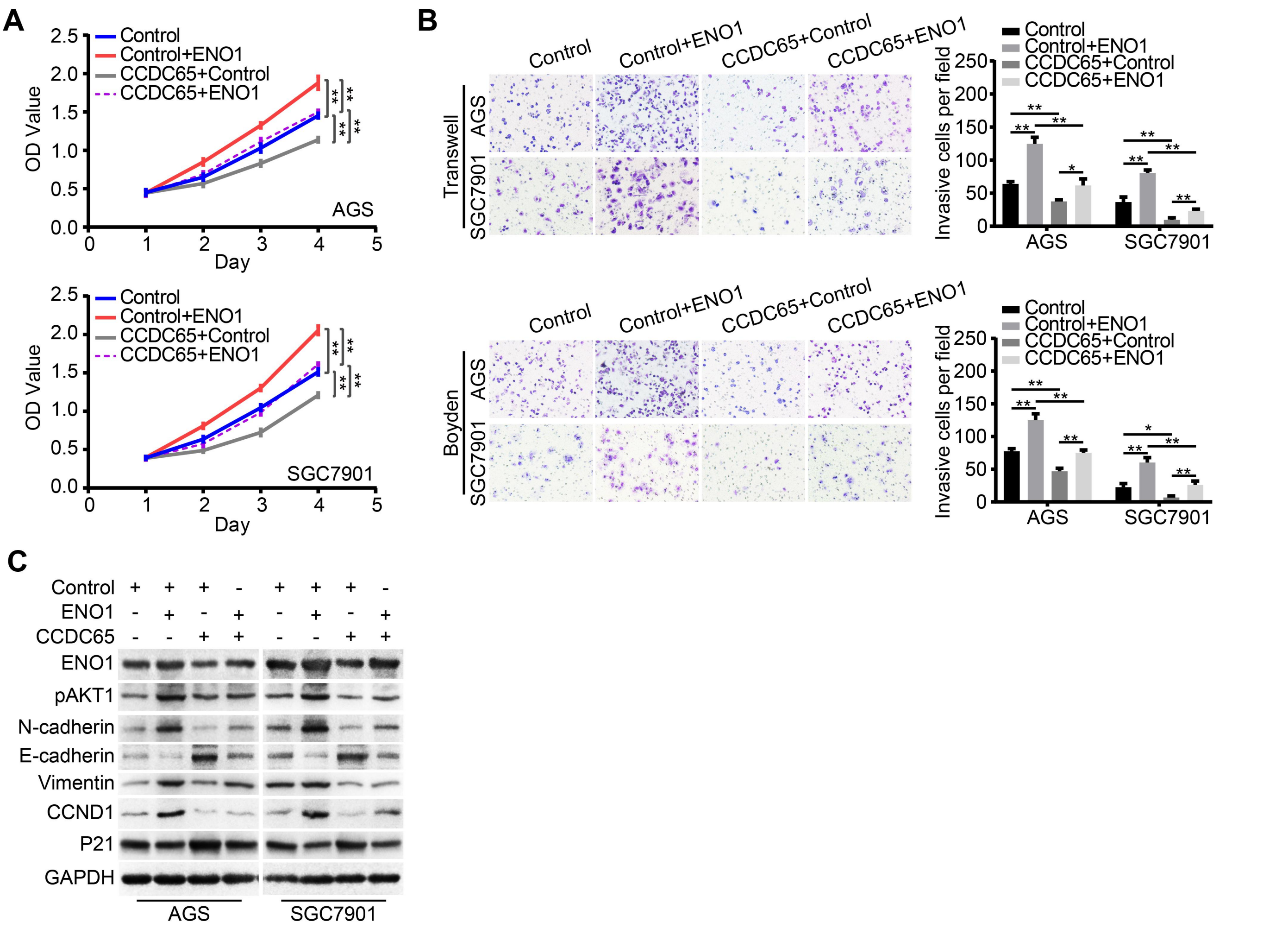

Supplementary Figure 4. Ectopic expression of ENO1 mitigates CCDC65 suppression of GC proliferation and metastasis. (A) MTT assays were used to detected the viability of GC cells in control, control+ENO1, CCDC65+control, CCDC65+ENO1 groups. **(B)** Transwell and boyden assay were carried out in control, control+ENO1, CCDC65+ control, CCDC65+ENO1 groups. **(C)** The expression of ENO1, p-AKT1 (Ser473), N-cadherin, E-cadherin, Vimentin, CCND1 and P21 in control, control+ENO1, CCDC65+ control, CCDC65+ENO1 groups.

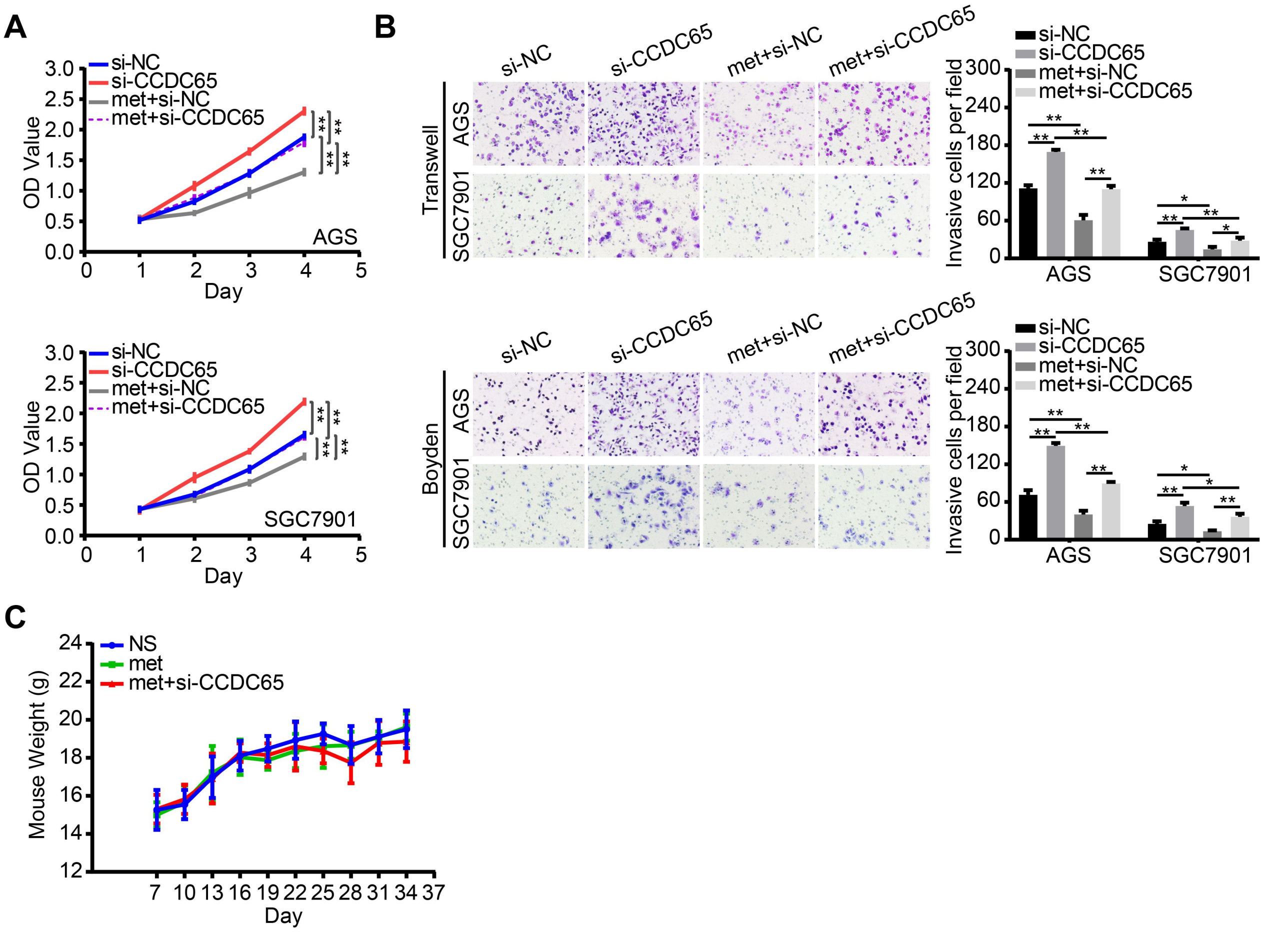

Supplementary Figure 5. Metformin potentiates CCDC65-mediated suppression of proliferation, metastasis and ENO1-AKT1 signal in GC. (A-B) MTT, transwell and boyden assay were carried out in si-NC, si-CCDC65, metformin+ si-NC, metformin+ si-CCDC65 groups. **(C)** The weight of mice treated with normal saline (NS), metformin (met) alone or metformin in combination with si-CCDC65 was collected every 3 days.

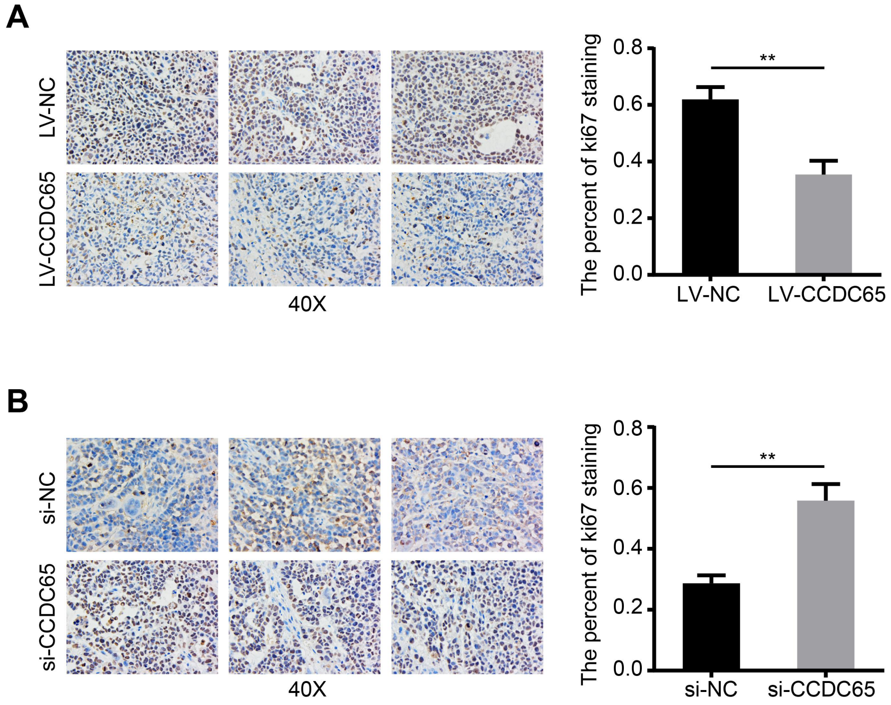

Supplementary Figure 6. The percent of ki67 staining of xenograft. (A) The percent of ki67 staining in xenograft tumors originating from LV-NC and LV-CCDC65 cells. **(B)** The percent of ki67 staining in xenograft tumors originating from si-NC and si-CCDC65 cells.

Supplementary Table 1： Association of CCDC65 expression with gastric cancer clinicopathological characteristics.

Parameters	Category	NO.	CCDC65 expression		P
			Low (97)	High (90)	
Age (years)					0.7118
	< 60	64	32	32	
	≥ 60	123	65	58	
Gender					0.7494
	Male	133	68	65	
	Female	54	29	25	
T stage					0.0012
	T1+T2	29	7	22	
	T3+T4	158	90	68	
N stage					0.0001
	N0+N1	93	35	58	
	N2+N3	84	62	22	
M stage					0.0157
	M0	157	75	82	
	M1	30	22	8	
Clincal stage (AJCC)					0.0006
	I+II	64	22	42	
	III+IV	123	75	48	

Supplementary Table 2: The sequences used in this study.

gene	No.	target sequences
CCDC65	1	GACTCACCCTGGAAAGTAA
	2	CCAGAGAACTTCATAAGGA
	3	CAAGGAGTTTGAGACAGAA
ENO1	1	AGTCCTTCATCAAGGACTA
	2	GCTGCTGAAGACTGCTATT
	3	GGAAGTATGACCTGGACTT
FBXW7	1	GGAACCCAAAGACCTGCTA
	2	GTTAGTGGTTCTGATGACA
	3	GCGTTGTATGCATCTTCAT

Supplementary Table 3: The primers used in this study.

Primers name		Sequence (5'-3')
CCDC65	Forward	AGAACTGTCCTTCGGGAAGTC
	Reverse	CTCGTTCAAATGTTTGGCTGAG
ENO1	Forward	GCCGTGAACGAGAAGTCCTG
	Reverse	ACGCCTGAAGAGACTCGGT
GAPDH	Forward	TGCACCACCAACTGCTTA
	Reverse	GGATGCAGGGATGATGTTC
ACTB	Forward	GACCTGACTGACTACCTCATGAAGAT
	Reverse	GTCACACTTCATGATGGAGTTGAAGG

Supplementary Table 4. A list of antibodies used for Western blot, IHC, IF, Co-IP.

antibody	Company	Cat.No	Mol weight (kDa)	Dilution
CCDC65	Proteintech	24376-1-AP	57	1:1000 (WB)<br>5 μg (IP)
CCDC65	Abcam	ab122482	57	1:50 (IHC)<br>1:100 (IF)
ENO1	Proteintech	11204-1-AP	47	1:5000 (WB)<br>1:250 (IHC)<br>1:100 (IF)
ENO1	CST	3810	47	1:50 (IP)
AKT1	SAB	48884	60	1:1000 (WB)<br>1:100 (IF)
Phospho-AKT1 (Ser473)	CST	9018	60	1:3000 (WB)<br>1:400 (IHC)
Nca	Proteintech	66219-1-Ig	130	1:2000 (WB)
Eca	Proteintech	60335-1-Ig	120	1:2000 (WB)
vimentin	Proteintech	10366-1-AP	54	1:1000 (WB)
CCND1	Proteintech	60186-1-Ig	36	1:5000 (WB)
P21	CST	2947	21	1:1000 (WB)
FBXW7	Proteintech	28424-1-AP	100-110	1:1000 (WB)<br>5 μg (IP)
ubiquitin	Proteintech	10201	8, 24	1:500 (WB)
PCNA	Proteintech	10205-2-AP	36-38	1:500 (IHC)
Ki67	Proteintech	27309-1-AP	-	1:8000 (IHC)
FLAG	Sigma	F1804	-	1:1000 (WB)<br>5 μg (IP)
MYC-tag	Proteintech	16286-1-AP	-	1:1000 (WB)<br>5 μg (IP)
HA-tag	Proteintech	51064-2-AP	-	1:3000 (WB)<br>5μg (IP)
GST	Proteintech	10000-0-AP	-	1:1000 (WB)<br>5 μg (IP)
His	Proteintech	66005-1-Ig	-	1:1000 (WB)<br>5 μg (IP)
GAPDH	Proteintech	60004-1-Ig	36	1:5000 (WB)
ACTB	Proteintech	66009-1-Ig	42	1:5000 (WB)

WB: western blot

IHC: immunohistochemistry

IF: immunofluorescence

Co-IP: co-immunoprecipitation
